# Caesarean section rates in a tertiary teaching hospital in northern Uganda: a retrospective analysis using the robson ten group classification system

**DOI:** 10.1186/s12884-024-06689-4

**Published:** 2024-07-20

**Authors:** Eric Ssennuni, Felix Bongomin, Elvis Akuma, Kizito Lukujja, Henry Kule, Keneth Opiro, Silvia Awor, Baifa Arwinyo, Sande Ojara, Jimmyy Opee, Ayikoru Jackline, Akello Jackline, Pebalo Francis Pebolo

**Affiliations:** 1 Faculty of Medicine, Gulu University, P.O. Box 166, Gulu, City Uganda; 2 Department of Obstetrics and Gynaecology, Gulu Regional Referral Hospital, Gulu City, Uganda; 3 College of Health Sciences, Makerere University, Makerere, Kampala Uganda

**Keywords:** Caesarean section rates, Robson ten group classification system, Tertiary teaching hospitals, Low-risk obstetric population

## Abstract

**Background:**

The Robson Ten Groups Classification System (RTGCS) is increasingly used to assess, monitor, and compare caesarean section (CS) rates within and between healthcare facilities. We evaluated the major contributing groups to the CS rate at Gulu Regional Referral Hospital (GRRH) in Northern Uganda using the RTGCS.

**Methods:**

We conducted a retrospective analysis of all deliveries from June 2019 through July 2020 at GRRH, Gulu city, Uganda. We reviewed files of mothers and collected data on sociodemographic and obstetric variables. The outcome variables were Robson Ten Groups (1–10) based on parity, gestational age, foetal presentation, number of foetuses, the onset of labour, parity and lie, and history of CS.

**Results:**

We reviewed medical records of 3,183 deliveries, with a mean age of 24.6 ± 5.7 years. The overall CS rate was 13.4% (*n* = 427). Most participants were in RTGCS groups 3 (43.3%, *n* = 185) and 1 (29.2%, *n* = 88). The most common indication for CS was prolonged labour (41.0%, *n* = 175), followed by foetal distress (19.9%, *n* = 85) and contracted pelvis (13.6%, *n* = 58).

**Conclusion:**

Our study showed that GRRH patients had a low-risk obstetric population dominated by mothers in groups 3 and 1, which could explain the low overall CS rate of 13.4%. However, the rates of CS among low-risk populations are alarmingly high, and this is likely to cause an increase in CS rates in the future. We recommend group-specific interventions through CS auditing to lower group-specific CS rates.

**Supplementary Information:**

The online version contains supplementary material available at 10.1186/s12884-024-06689-4.

## Introduction

Caesarean section (CS), a life-saving surgical intervention in obstetrics, should be universally accessible because of its known reduction in maternal and neonatal morbidity and mortality [1]. Globally, a rate of 21.1% was realized in 2021, low-income countries have rate of 8.2%, and middle-income countries have a rate of 24.2% [2]. The global CS rate is projected to increase to 28.5% by the year 2030 [3]. In sub-Saharan African countries, where maternal and perinatal mortality are high, CS rate is lower (7.3%) than that in the least developed countries (8.2%) [2]. In 2015, the World Health Organization (WHO) recommended that ‘*every effort should be made to provide CS to women in need*,* rather than striving to achieve a specific rate”* [4].

WHO cautions that a population or facility CS rate below 5% suggests a lack of access to emergency obstetric care services while rate between 10 and 15% is generally accepted as the optimal range. This WHO expert opinion was based on limited data mainly from Northern Europe with quality healthcare setups [5]. Tremendous global increase in CS rates has been seen in recent years, especially among high-income countries, raising concerns about over-utilization of CS without added benefits [6]. There is no evidence showing the benefits of caesarean delivery for mothers or babies who do not require the procedure [5]. The high cost associated with CS may lead to unnecessary expenditures for already overburdened and economically hard-hit families, especially in low-income countries [7]. Although the WHO recommends no specific CS, there are increasing rates of unjustifiable CS, with up to 34.9% having no clear indication in a Chinese prospective cohort study [8]; moreover, CS audit intervention reduced unnecessary CS in up to 52% of patients in Tanzania [9].

The Robson Ten Group Classification System (RTGCS) is a simple method that provides a common starting point for further detailed analysis, within which all perinatal events and outcomes can be measured and compared [10]. In 2015, the WHO published a statement proposing the RTGCS for assessing, monitoring and comparing CS rates within and across healthcare units over time [10]. It can work as an audit intervention to identify target groups that have the greatest impact on the CS rate [10] and to assess, monitor and compare rates both within and between healthcare facilities over time (FIGO, 2016). It also helps to develop a strategy for reducing the CS rate if it is found to be unjustifiably high [11].

The RTGCS classifies all mothers delivering from a particular facility into 10 groups using six routinely collected obstetric variables, namely, parity, presence of previous caesarean scar, onset of labour, number of foetuses, gestational age, and foetal lie/presentation [5]. It is a robust, clinically relevant, simple, easy to use and easily reproducible tool [12, 13].

In Uganda, the CS rates stand at 6% and are seen to be higher 11% among first-order births indicating a high incidence of primary CS [14]. Despite the low rate of CS in Uganda, there are massive variation in the facility CS rates across the country. The average tertiary facility CS rates in Uganda was 32% in the financial years 2018/2019 yet Gulu Regional Referral Hospital (GRRH) rate has lowest (14%)in the same period [15].The low CS in GRRH and the marked differences in CS rates among facilities in Uganda and even within Northern Uganda could best be explored using RTGCS. However, despite its popularity, we could not find any studies or reports of the tools that have been used to study the trend of CS in any of the facilities in Northern Uganda except in a private non for profit-based hospital, in Kampala, the capital of Uganda [16]. Therefore, this study aimed to identify the major contributing groups to CS rates at an urban tertiary teaching hospital in Northern Uganda.

## Methods

### Study design

This was a cross-sectional retrospective chart review conducted at GRRH in Gulu city, Northern Uganda, between July 2019 and June 2020.

### Study setting

GRRH is a tertiary health care facility with a total annual number of deliveries of approximately 4,000-4500 (from ward records). It is a public hospital that serves as a teaching hospital for Gulu University, an internship training centre for medical, nursing, midwifery and pharmacy graduates. Recently, it has been accredited as a fellowship training centre for the East Central and Southern African College of Obstetrics and Gynaecology (ECSACOG). It is a referral site for more than 8 districts in Northern Uganda, serving a population of approximately 2 million people.

### Study population

The study population included all women who delivered at the facility during the study period. We excluded mothers who underwent exploratory laparotomy due to uterine rupture. We used the Uganda Ministry of Health 2016 Essential Maternal and Neonatal Clinical Care Guidelines, in which viability is considered after 28 weeks of gestation or a foetal birth weight of 1000 g or more. This is because our study period was before the newly revised 2022 guidelines that reduced the cut-off for viability to 26 weeks [17].

### Data source and variables

The data were collected by trained research assistants using a structured data extraction template on the Open Data Kit (ODK) for an android platform and saved on a central server. These research assistants were trained record assistants who work within the record office in GRRH. The maternity ward’s patient admission records for one Uganda Government financial year were collected and examined for detailed obstetric information. This information included previous obstetric history (parity and previous CS), labour onset (spontaneous, induced, or CS before labour), foetal presentation or lying (cephalic, breech, transverse, or oblique), number of foetuses (single or multiple), delivery method (vaginal or CS), and gestational age (term or preterm).

The gestational age was retrieved from the files that were calculated by either the last menstrual date or by an ultrasound scan performed before 30 weeks. For those with ultrasound scans after 30 weeks and/or with no records of gestational estimation based on last normal menstrual dates, foetal birthweights were used as a proxy to estimate gestational age. A birth weight < 2,500 g was considered preterm, and a birth weight ≥ 2,500 g was considered term. This method was used elsewhere to estimate foetal weight [18]. The indications for CS was taken from the surgeon’s operation notes or the clinical review notes. For cases were all this information is missing, the indications is indicated as “missing”.

### Study measurements

The outcome variables were Robson groups (1–10) based on parity, gestational age, foetal presentation, number of foetuses, onset of labour and history of CS (supplementary file 2; RTGCS Table).

### Data processing and analysis

The data were exported, cleaned and analysed using IBM SPSS Statistics for Windows, version 29 (IBM Corp., Armonk, N.Y., USA). The caesarean section rate was calculated as the total number of caesarean sections divided by the total number of deliveries in the study period. Women were categorized into one of the ten Robson groups, and 9 women had no information relating to their parity and categorized as unclassified. The caesarean section rate was calculated for each obstetric population, and its contribution to the overall caesarean section rate was calculated.

## Results

### General characteristics of women who gave birth at GRRH, July 2019-June 2020

During the one-year period, files of 3183 mothers who delivered at GRRH were retrieved and included in the final analysis. The mean age of the participants was 24.6 years (SD 5.7).

As summarised in table Table 1  below, para 1-4 contributed to more than half of the total participants 58.1%, (*n* = 1847), most pregnancy was at term 89.9%, (*n* = 2861) with singleton 98.5% (*n* = 3136) and cephalic presentation 95%(*n* = 3024), and 4.5% (*n* = 142) had breech. Most had spontaneous onset of labour 97.9%, (*n* = 3116) The rate of CS was 13.4% (*n* = 427). One in ten 10.1% (*n* = 322) of the mothers were referred to the facility. Two thirds of the babies had normal birthweights of 2-5-3.5 kg (66.9%, *n* = 2128). Nearly 1 out of 5 or 17.7% (*n* = 563) of the women who delivered at GRRH did not have clear documentation of their HIV results in their files; among those who did, up to 16.3% (*n* = 519) were documented as HIV positive (RTGCS 1).

**Table 1:** Characteristics of women who gave birth at GRRH between July 2019 and June 2020

Characteristics	Frequency(*N*)	Mean(*n*)/Percentage (%)
Maternal Age (mean; SD) Min–Max	3157	24.58(5.71)13–48
**Parity**		
0	1131	35.5
1–4	1847	58.1
5 and Above	194	6.2
Not Recorded	11	0.3
**Gestational Age**		
Preterms (< 37 weeks)	322	10.1
Terms (≥ Weeks)	2861	89.9
**Uterine Scar Status**		
With Uterine Scar	62	1.9
No Uterine Scar	3121	98.1
**Onset of labor**		
Spontaneous	3116	97.9
Induced	36	1.1
Caesarean Section Before Labor Onset	31	1.0
**Presentation**		
Cephalic	3024	95
Breech	142	4.5
Transverse/Obligue Lie	17	0.5
**Mode of delivery**		
SVD	2638	82.9
CS	427	13.4
AVD	27	0.8
VBD	91	2.9
**Fetal Outcome**		
Live Birth	3021	94.9
Stillbirth	95	3.0
Admission to NICU	67	2.1
**Birth Weight**		
Very Low Birthweight (1–1.5 kg)	20	0.6
Low Birthweight (1-0.6-2.4 kg)	227	7.1
Normal Birthweight (2.5–3.5 kg)	2128	66.9
Big baby (3.5 or more	704	22.1
Not recorded	104	3.3
**Number of Fetus**		
Singleton	3136	98.5
Multiple	47	1.5
**Gender**		
Female	1404	44.1
Male	1779	55.9
Referral in Patient		
Attendant At GRRH	2861	89.9
Referred to GRRH	322	10.1
**HIV Status**		
Positive	519	16.3%
Negative	2101	66%
Unknown	563	17.7%

**AVD** = assisted vaginal delivery; **CS** = caesarean section; **SVD** = spontaneous vaginal delivery; **VBD** = vaginal breech delivery; **NICU** = Neonatal Intensive Care Unit; **GRRH** = Gulu Regional Referral Hospital; **SD**, = Standard Deviation

### The Robson Ten groups and their relative contributions to the overall CS rate

Women in Group 3 made the largest contribution to the obstetric population, accounting for 54.0% (*n* = 1719) of all deliveries. This was followed by Group 1 and Group 10, which accounted for 29.0% (*n* = 923) and 7.9% (*n* = 252), respectively. The fourth largest group, 2.9% (*n* = 93), was Group 7. Nine (9) women could not be classified into any of the ten groups due to missing parity information in the files (Table 2).

**Table 2:** Proportion of each Robson group, CS rate in each group, and their relative and absolute contributions to the overall CS rate at GRRH

Group definition	Number of CS in the group *N*	Size of Group *N* (%) ^a^	Group CS rate (%) ^b^	Absolute group contribution to overall CS (13.4%) ^c^	Relative group contribution to overall CS (%^) d^
Group 1	88	923(29)	9.5	2.76	20.61
Group 2^e^	6	16 (0.5)	37.5	0.19	1.41
Group 3	185	1719(54)	10.8	5.81	43.32
Group 4^f^	10	18(0.6)	55.6	0.31	2.34
Group 5	33	52(1.6)	63.5	1.04	7.73
Group 6	10	39(1.2)	25.6	0.31	2.34
Group 7	35	93(2.9)	37.6	1.10	8.20
Group 8	14	46(1.4)	30.4	0.44	3.28
Group 9	10	16(0.5)	62.5	0.31	2.34
Group 10	35	252(7.9)	13.9	1.10	8.20
Unclassified	1	9(0.3)	11.1	0.03	0.23
	427	3183(100)			100

**CS** = caesarean section; **GRRH** = Gulu Regional Referral Hospital

^**a**^ Group size (%) = n of women in the group/total N women delivered in the hospital × 100, ^**b**^ Group CS rate (%) = n of CS in the group/total N of women in the group × 100, ^**c**^ Absolute group contribution (%) = n of CS in the group/total N of women delivered in the hospital × 100, ^**d**^ Relative group contribution (%) = n of CS in the group/total N of CS in the hospital × 100, ^**e**^ Groups 2a and 2b were merged into one group due to their relatively small size, ^**f**^ Groups 4a and 4b were merged into one group due to their relatively small size

The largest contributors to the overall CS rate were Group 3 (43.3%, *n* = 185) and Group 1 (20.6%, *n* = 88). These two groups contributed approximately 63.9% (*n* = 273) of all caesarean deliveries at GRRH. Groups 7 and 10 each contributed 8.2% (*n* = 35) of the total CSs, while group 5 (those with at least one previous caesarean scar, singleton, cephalic pregnancy at term) was the fifth largest contributor to the overall CS, accounting for 7.7% (*n* = 33) (Table 2).

### Caesarean section across the RTGCS

Sub analysis of the indications for CS showed that prolonged labour was the most common indication, accounting for up to 41.0% (*n* = 175) of the total CS performed in this study. These were followed by foetal distress and contracted pelvis at 19.9% (*n* = 85) and 13.6% (*n* = 58), respectively. Up to 7.7% (*n* = 33) of the mothers had no clear indication for CS recorded in their files (Table 3). Up to 11.9% (22) of 185 cases in group 3 had their indications documented as contracted pelvis.

**Table 3:** Indications for caesarean section in GRRH across the RTGCS

		CS counts												
RTGCS Category		G 1	G 2	G 3	G 4		G 5	G 6	G 7	G 8	G 9	G 10	Gx**	Total N (%)
Indication of CS	APH	4	1	5		0	2	1	3	2	1	2	0	21(4.9)
	Breech	0	0	0		0	0	6	1	0	0	0	0	7(1.6)
	Contracted Pelvis	12	1	22		3	0	0	3	3	4	10	0	58(13.6)
	Eclampsia	1	0	2		0	0	1	0	0	0	1	0	5(1.2)
	Foetal distress	14	0	57		0	0	2	2	0	0	10	0	859(19.9)
	Foetal macrosomia	0	0	4		0	1	0	0	0	0	0	0	5(1.2)
	Malpresentation	0	1	4		0	1	0	1	1	0	0	0	8(1.9)
	Indication Missing	5	0	18		1	2	0	0	0	2	5	0	33(7.7)
	Previous CS	0	0	0		0	16	0	11	1	0	2	0	30(7.0)
	Prolonged labour	52	3	73		6	11	0	14	7	3	5	1	175(41.0)
Total N (%)		88 (20.61)	6 (1.41)	185 (43.32)		10 (2.34)	33 (7.73)	10 (2.34)	35 (8.20)	14 (3.28)	10 (2.34)	35 (8.20)	1 (0.23)	427 (100)

**Gx Unclassified, **G1-G10**. Group 1 through Group 10; **APH** = antepartum haemorrhage; **RTGCS** = Robson Ten Group Classification System; **CS** = caesarean section; **GRRH** = Gulu Regional Referral Hospital

### Mode of delivery for mothers with previous uterine scar(s)

Among the 62 mothers who had previous uterine scars, 54.8% (*n* = 34) had only one previous uterine incision scar. Moreover, of the 28 mothers who had more than one previous uterine scar, 14.3% (*n* = 4) had successful vaginal births after CS (VBACs), one had assisted vaginal birth, 2 had assisted breech deliveries, and one had spontaneous vaginal birth. Of those who had one previous uterine incision, 24 of the 34 underwent repeat CS (Table 4).

**Table 4:** Mode of delivery for mothers with previous uterine scar(s) in *GRRH*

Previous Uterine Scar and Mode of Delivery					
			Previous Uterine Scar		Total n (%)
			More than one Previous Uterine Scar *N* = 28 (45.2%)	One Previous Uterine Scar *N* = 34 (54.8%)	
Final mode of delivery	AVD	Count	1(1.6)	0	1(1.6)
	CS	Count	23(37.1)	24(38.7)	47(75.8
	SVD	Count	3(4.8)	9(14.5)	12(19.4)
	VBD	Count	1(1.6)	1(1.6)	2(3.2)

**AVD** = assisted vaginal delivery; **CS** = caesarean section; **SVD** = spontaneous vaginal delivery; **VBD** = vaginal breech delivery, **GRRH** = Gulu Regional Referral Hospital

## Discussion

The determinants of rising CS trends worldwide are controversial and are sometimes blamed on nonmedical indications [19]. Quality assurance in CS is needed to reduce maternal and perinatal morbidity and mortality, especially in low-resource settings such as Uganda. Analysis of CS trends using the RTGCS provides a common starting point for further detailed audit, within which all perinatal events and outcomes can be measured and compared to inform such quality assurance interventions.

With a retrospective study design to assess 3183 women who delivered at GRRH during the study period, we used the RTGCS to assess the proportion of each group in the obstetric population, the contribution of CS in each group to the overall CS rate and the CS rate within each group. Approximately 9 (0.3%) of these women could not be classified due to missing information on parity in their files. Also, up to 11.9% (22) participants in group 3, who have had successful vaginal birth had their CS indication documented as contracted pelvis. This shows the challenges with retrospective data in clinical practices in resource limited settings and call for routine data collection and vetting.

Our study revealed that more than three-quarters of the mothers who gave birth at GRRH belonged to groups 3 and 1, representing a low-risk obstetric population. This is similar to a findings in a study done in Kampala, the capital of Uganda [16] and in the neighbouring Tanzania and Ethiopia [20, 21]. The low CS rates at GRRH can be attributed to the fact that most of the mothers belong to the low-risk obstetric population groups 3 and 1. Furthermore, the high-risk obstetric population, including groups 5, 9, and 10, accounted for only 10% of the total number of mothers who delivered during the study period. The current overall CS rate in this study is comparable to the 14% reported in the previous fiscal year of 2018/2019, and it is lower than one-third of the average CS rate in tertiary hospitals in Uganda [15] confirming earlier suggestions that the low CS rates in GRRH patients are justifiable [22].

Despite groups 3 and 1 being classified as low-risk populations, one in ten mothers in these groups underwent CS, accounting for 63.9% of all caesarean deliveries at GRRH during the study period. Similarly, a study conducted in a tertiary hospital in Ethiopia identified group 3 as the primary contributor to the overall CS rate [21]. In contrast, a study done in a tertiary non for-profit faith-based hospital in Kampala showed that group 5 was the leading contributor to CS with a rate of 35.4% while group 3 was the third contributor to the overall CS rates (13.7%). Our study, however, indicated that group 1 was the second major contributor to the overall CS rate similar to a finding in Kampala [16], while a similar study in Ethiopia identified group 5 as the second significant contributor [21]. Similarly, in a study at a rural hospital in Tanzania, group 1 was the largest contributor to the overall CS rate, followed by group 3 [20]. These variations may be attributed to differences in the obstetric population and the overall distribution of CS rates across all ten groups [4]. Nevertheless, this suggests high rates of CS, particularly among multiparous mothers with no previous history of uterine scar, which are likely to reduce the likelihood of future vaginal deliveries among mothers delivering at this facility.

The high CS rate among mothers in group 5 in our study aligns with similar findings in other studies conducted in low-income countries and it has been identified as a primary contributing factor in several studies [16, 20, 21, 23]. Although the group’s contribution to the overall CS rate appears to be negligible, this is attributed to the small obstetric population, which may increase in the future due to rising CS rates among the low-risk population [24]. Of particular interest in this study are the CS rates among mothers with a single previous uterine scar, with close to three-quarters of these mothers undergoing repeat CS. Although it is challenging to discern the indications for repeat CS in this retrospective study, the high rates suggest that most mothers in this subgroup were not given the opportunity for a vaginal birth after caesarean section (VBAC). This finding is consistent with a prospective cohort study in Ethiopia, where up to 62% of mothers with one previous uterine incision had repeat CS [18].

The CS rate in group 10 was lower than that in the reference population of a multicounty survey conducted by Tognon et al., 2019 [20], indicating that mothers in this group had higher rates of spontaneous vaginal deliveries [4]. However, group 10 still contributed significantly to the overall CS rate due to its relatively high-risk population. It is important to note that the CS rate among group 9 in our study did not reach 100% as per the RTGCS reference guide [4].

Several limitations should be noted in this study. The retrospective design with data capture, especially from clinical records, may have led to misallocated cases as well as indications. Data on partograph to chart progress and aids in the diagnosis of labour dystocia are not often documented questioning the primary cause of prolonged labour in this study. Additionally, the definition of foetal viability based on 28 weeks from the last normal menstrual period or an ultrasound scan before 30 weeks, if the last normal menstrual period date is missing, or foetal weight of 1000 g or more could impact case allocation in the groups. This finding may not be generalizable to countries with different viability cut-offs, including the current setting in Uganda, where viability has changed from 28 weeks to 26 weeks according to the new guidelines [17]. Despite these limitations, the findings in this study serve as a starting point to question why a particular group has a high CS rate rather than solely focusing on the rate of this obstetric procedure.

## Conclusions

Our study revealed that GRRH patients had a low-risk obstetric population, primarily consisting of mothers in groups 3 and 1, which may account for the low overall CS rate of 13.4%. However, the high rates of CS among this low-risk group are concerning and are likely to lead to an increase in CS in the near future. It is important to develop group-based interventions to address these issues. We recommend implementing group-specific interventions, such as conducting a caesarean section audit among the low-risk obstetric population, to reduce group-specific CS rates. The adoption of RTGCS in all facilities can help monitor group-specific rates, providing valuable information for targeted interventions, as opposed to relying solely on general CS rates, which can be misleading. A prospective study with routine data verification involving multiple tertiary and general hospitals in the country could be essential for understanding and designing auditing interventions to combat the increasing prevalence of primary CS.

### Electronic supplementary material

Below is the link to the electronic supplementary material.

Supplementary Material 1

Supplementary Material 2

## Data Availability

Supplementary file included with the submission.
