# Supplementary material for: Caesarean section rates in a tertiary teaching hospital in northern Uganda: a retrospective analysis using the robson ten group classification system
Source: BMC Pregnancy Childbirth. 2024 Jul 20;24:489. doi: 10.1186/s12884-024-06689-4 (PMC11264903; doi:10.1186/s12884-024-06689-4)
Supplement: Supplementary file 2 — Supplementary Material 2 [file 12884_2024_6689_MOESM2_ESM.docx]

| Group | Description | |
| --- | --- | --- |
| 1 | Nulliparous, singleton, cephalic, ≥37 weeks’ gestation, in spontaneous labor | |
| 2 | Nulliparous, singleton, cephalic, ≥37 weeks’ gestation, induced labour or caesarean section before labour | |
| 2 | a | Nulliparous, singleton, cephalic, ≥37 weeks’ gestation, induced labour |
|  | b | Nulliparous, singleton, cephalic, ≥37 weeks’ gestation, caesarean section before labour |
| 3 | Multiparous (excluding previous caesarean section), singleton, cephalic, ≥37 weeks’ gestation, in spontaneous labour | |
| 4 | Multiparous without a previous uterine scar, with singleton, cephalic pregnancy, ≥37 weeks’ gestation, induced or caesarean section before labour. | |
| 4 | a | Multiparous without a previous uterine scar, with singleton, cephalic pregnancy, ≥37 weeks’ gestation, induced labour |
|  | b | Multiparous without a previous uterine scar, with singleton, cephalic pregnancy, ≥37 weeks’ gestation, caesarean section before labour |
| 5 | Previous caesarean section, singleton, cephalic, ≥37 weeks’ gestation | |
| 6 | All nulliparous with a single breech | |
| 7 | All multiparous with a single breech (including previous caesarean section) | |
| 8 | All multiple pregnancies (including previous caesarean section) | |
| 9 | All women with a single pregnancy in transverse or oblique lie (including those with previous caesarean section) | |
| 10 | All singleton, cephalic, <37 weeks’ gestation pregnancies (including previous caesarean section). | |

**Appendix; World Health Organisation Robson ten-group delivery classification system**
